# Radiofrequency Ablation for Cervical Metastatic Lymph Nodes in Children and Adolescents With Papillary Thyroid Carcinoma: A Preliminary Study

**DOI:** 10.3389/fendo.2021.624054

**Published:** 2021-05-18

**Authors:** Lin Yan, Ying Zhang, Bo Jiang, Yukun Luo

**Affiliations:** Department of Ultrasound, First Medical Center, Chinese People’s Liberation Army (PLA) General Hospital, Beijing, China

**Keywords:** papillary thyroid carcinoma, radiofrequency ablation, children, adolescents, metastatic lymph node

## Abstract

**Objective:**

To evaluate the safety and efficacy of radiofrequency ablation (RFA) for metastatic lymph nodes (LNs) in children and adolescents with papillary Thyroid Carcinoma (PTC).

**Materials and Methods:**

From December 2014 to March 2018, 10 metastatic LNs(mean volume 0.30 ± 0.38 ml, range 0.06-1.23ml) in 5 children and adolescents (3 females, 2 males; mean age 15.60 ± 2.97 years, range 12-19 years) with PTC treated by RFA were evaluated in this study. The mean number of surgical procedures performed before RFA was 1.2 (range 1-2) and the mean number of treated metastatic LNs per patient was 2 (rang 1-3). RFA was performed with an 18–gauge bipolar RF applicator under local anesthesia. Follow-up consisted of US and serum thyroglobulin (Tg) level at 1, 3, 6, 12 months and every 12 months thereafter.

**Results:**

All the patients were well tolerant to RFA procedure and no procedure-related complications occurred. During a mean follow-up time of 52.00 ± 21.44 months, the initial volume of LNs was 0.30 ± 0.38 ml, which significantly decreased to 0.01 ± 0.03 ml (*P* = 0.005) with a mean VRR of 99.28 ± 2.27%. A total of 9 metastatic LNs (90.00%) completely disappeared. After RFA, 2 patients developed newly metastases. One patient had additional RFA. The other one with multiple LN metastases underwent total thyroidectomy with central neck dissection.

**Conclusion:**

As a less invasive and effective technique, RFA may provide another alternative to the existing therapeutic modalities for cervical metastatic LNs in children and adolescents with PTC.

## Introduction

Differentiated thyroid cancer (DTC) is the most common pediatric endocrine malignancy, accounting for 1% of all cancers in prepubertal children and up to 7% in adolescents, with a rising incidence in the pediatric population over the last decade (1). The most common subtype of DTC in pediatric patients is papillary thyroid carcinoma (PTC). It is generally agreed that the clinical presentation of PTC in pediatric patients is different from that in adults (2). The prognosis is excellent, but pediatric patients usually present in the advanced stage with a large size at diagnosis and the incidence of local or distant recurrence is much higher than their adult counterparts (2–5). The majority of recurrence is identified in cervical lymph node (LN) (2). The American Thyroid Association (ATA) guideline for children with thyroid nodules and DTC recommended three treatments for cervical metastatic LNs, which were repeat surgery, thyroid-stimulating hormone (TSH) suppression and ^131^ I therapy (2). Although repeat surgery was preferable to other treatments for the macroscopic cervical disease, finding a lesion in the neck intraoperatively could be difficult (6). For patients with small-volume cervical tumor, observation with TSH suppression could be considered, but there were no data to weigh the potential benefits against the potential risks of long-term suppression therapy for children and adolescents (2). ^131^ I therapy was another option for small-volume cervical tumor. Unfortunately, it could increase the risks of complications and secondary malignancies (6). Accordingly, in children and adolescents, it may be reasonable or appropriate to consider less invasive alternatives than repeated surgery and ^131^ I therapy.

Radiofrequency ablation (RFA), as a minimally invasive treatment, has been used as an alternative to surgery for various solid tumors in adults (7–9). It has been recommended as a safe and effective treatment for benign thyroid nodules and recurrent thyroid cancers (10). However, for children and adolescents, RFA was only considered as a treatment of choice for osteoid osteoma (11–13). There were limited experiences of thermal ablation in other organs or tissues (11, 14–18). Nevertheless, as less invasive and feasible treatments, thermal ablation techniques were promising alternatives for pediatric tumors (19). To date, there have been no studies on the clinical application of RFA for metastatic LNs in children and adolescents with PTC.

Therefore, the aim of this study was to evaluate the safety and efficacy of RFA for metastatic LNs in children and adolescents with PTC.

## Materials and Methods

The study was approved by the Institutional Review Board of Chinese PLA General Hospital. Written information consent was obtained from all the patients’ parents prior to RFA procedure.

### Patients

Between December 2014 to March 2018, 5 patients (3 females, 2 males, mean age 15.60 ± 2.97 years, range 12-19 years) with 10 metastatic LNs from PTC were treated by RFA in our institution. Inclusion criteria were: (1) age ≤ 20 years; (2) prior resection of primary tumor with pathological PTC confirmation; (3) patients with metastatic LNs confirmed by ≥ 2 separate fine-needle aspiration (FNA) or core needle biopsy (CNB); (4) the number of metastatic LNs was ≤ 3; (5) LNs deemed technically treatable based on US imaging and patient’s condition.

### Pre-Ablation Assessment

US examinations before and after RFA, as well as during follow-up were performed using a Siemens Acuson Sequoia 512 Ultrasound System (Siemens, Mountain View) with a 15L8W linear array transducer or a Philips iU22 Ultrasound System (Philips Healthcare) with a L12-5 linear array transducer or a Mindray M9 Ultrasound System (Mindray) with a L12-4 linear array transducer.

Before RFA, all the metastatic LNs were evaluated by US including the location, size, echogenicity, component and vascularity. For each metastatic LN, the diameters in three dimensions (the largest diameter and two perpendicular diameters) were recorded. The volume was calculated with the equations (10):

V=πabc/6

V is the volume, while a is the largest diameter, b and c are the other two perpendicular diameters.

### Ablation Procedures

All RFA procedures were performed by an experienced US physician with more than 20-year experience in thyroid US and interventional US. A bipolar RFA generator (CelonLabPOWER, Olympus Surgical Technologies Europe) and an 18–gauge bipolar RF applicator with 0.9 cm active tip was used (CelonProSurge micro 100-T09, Olympus Surgical Technologies Europe) in this study. During the application of RF energy, the generator continuously measured the electric impedance of the tissue between the two electrodes at the tip of the RF applicator. The power was automatically adjusted based on the change of tissue impedance.

RFA was performed in outpatient department. Patients were supine with the neck extended during the procedure. An IV line was introduced *via* the antecubital vein. Before RFA, in order to design the best insertion way, US and CDFI were performed by the operator to evaluate the relationship between metastatic LNs and cervical critical structures such as trachea, vessels, esophagus and recurrent laryngeal nerves. Local anesthesia with 1% lidocaine was injected at the subcutaneous puncture site and the periphery of metastatic LNs. RFA was performed using hydrodissection technique. Normal saline was injected using another needle (23 gauge) to separate the metastatic LN from critical structures in order to prevent thermal injury. The RFA power was 3 W, if a transient hyperechoic zone did not form at the electrode tip within 5–10 s, the radiofrequency power was increased to 5-8 W. The ablation was terminated when all portions of the target ablation area had changed to transient hyperechoic zones.

During the procedure, we gave special attention to the preservation of critical cervical structures in order to prevent significant complications such as hematoma or nerve injury. After ablation, each patient was observed for 1–2 hours in the hospital while any complication occurring during and immediately after ablation was carefully evaluated according to the clinical signs and symptoms.

### Post-Ablation Assessment

Clinical follow-up consisted of US and serum thyroglobulin (Tg) levels at 1, 3, 6 and 12 months and every12 months thereafter. The ablated volume, the largest diameter, vascularity and the development of new metastatic tumors were evaluated during the follow-up. The percentage volume reduction ratio (VRR) was calculated as follows:

$$VRR=\frac{\left(\text{initial}\;\text{volume}\;-\;\text{final}\;\text{volume}\right)}{\text{initial}\;\text{volume}}\times 100\%$$

Complications during follow-up were assessed using the reporting standards of the Society of Interventional Radiology (20, 21). The development of cervical metastatic LNs was evaluated by using criteria from ATA guideline for children with thyroid nodules and DTC (2), and suspicious lesions were submitted to biopsy.

### Statistical Analysis

Statistical analysis was performed using the SPSS statistical software (Version 25.0). Continuous data was expressed as mean± SD (range). Changes of the mean volume and diameter were compared using Wilcoxon signed rank tests before RFA and at the last follow-up visit. A difference with *P* < 0.05 was considered as statistically significant.

## Results

### Clinical Characteristics

Clinical characteristics of the 5 patients (3 females and 2 males) before RFA are summarized in **Table 1**. The mean age was 15.60 ± 2.97 years (range 12-19 years). All the patients had prior surgical history for PTC. Patient 1 had right lobectomy with isthmectomy. Patient 2 had subtotal thyroidectomy. Patient 3 had left lobectomy with isthmectomy initially and then central neck dissection because of cervical metastatic LNs. Patient 4 and Patient 5 had total thyroidectomy with central neck dissection and subsequent ^131^ I therapy. The mean number of surgical procedures performed before RFA was 1.2. The mean interval time between initial surgery and RFA was 3.20 ± 1.64 years (range 2-6 years). The mean number of treated metastatic LNs per patient was 2. The locations of metastatic LNs were as followed: 2 at right level IV, 5 at left level III, 2 at left level IV and 1 at right level VI. The mean of largest diameter of metastatic LNs was 1.16 ± 0.52 cm. The mean initial volume was 0.30 ± 0.38 ml.

**Table 1:** The clinical characteristics of the pediatric patients before RFA.

No. of patient	Sex/Age	Type of initial surgery/interval time (years)	^131^ I therapy	Recurrence beyond neck	No. of LN	Location/level	Largest diameter (cm)	Initial Volume (ml)
1	F/13	Right lobectomy with isthmectomy/2	No	No	1	Right/IV	0.7	0.06
					2	Right/IV	0.6	0.06
2	M/12	Subtotal thyroidectomy/2	No	No	3	Left/III	1.5	0.35
					4	Left/III	0.9	0.08
3	F/17	Left lobectomy with isthmectomy/6; Central neck dissection/2	No	No	5	Left/III	1.6	0.68
					6	Left/III	0.6	0.06
4	M/17	Total thyroidectomy with central neck dissection/3	Yes	Lung	7	Left/IV	2.0	1.23
					8	Left/IV	1.8	0.28
					9	Left/III	0.9	0.07
5	F/19	Total thyroidectomy with central neck dissection/3	Yes	No	10	Right/VI	1.0	0.16

### RFA Procedure and Complications

A power of 3W was used in 4 patients and 6W was used in 1 patient. The mean ablation time was 158.80 ± 65.13s (range 98-263s), and the mean energy was 522.00 ± 211.59 J (range 290-760J). All the patients were well tolerable the RFA procedure. None of the patients experienced any life-threatening or delayed complications related to RFA during the follow-up.

### Follow-Up

**Figure 1:**
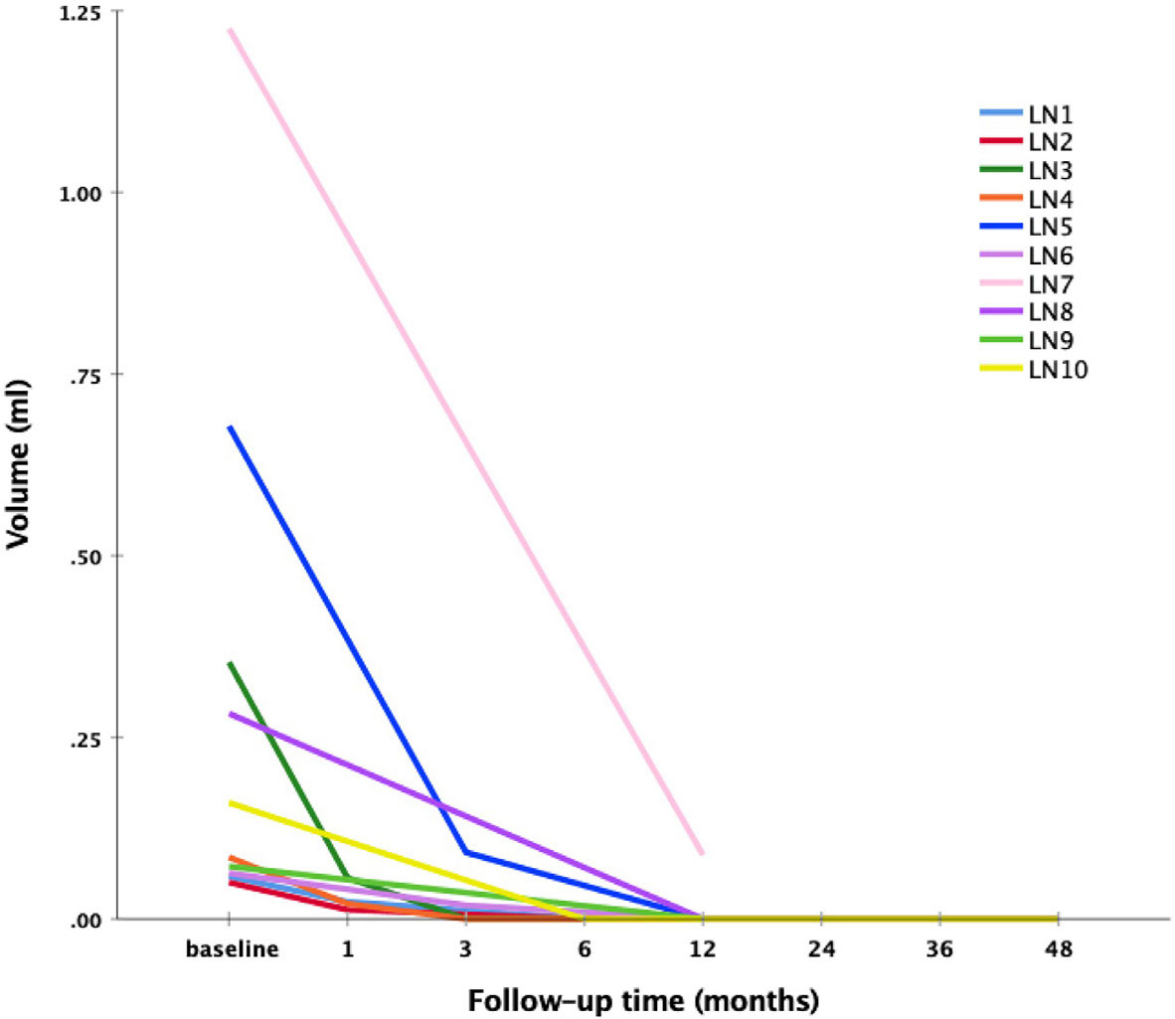
The volume changes of ablated LN after RFA at follow-up.

**Figure 2:**
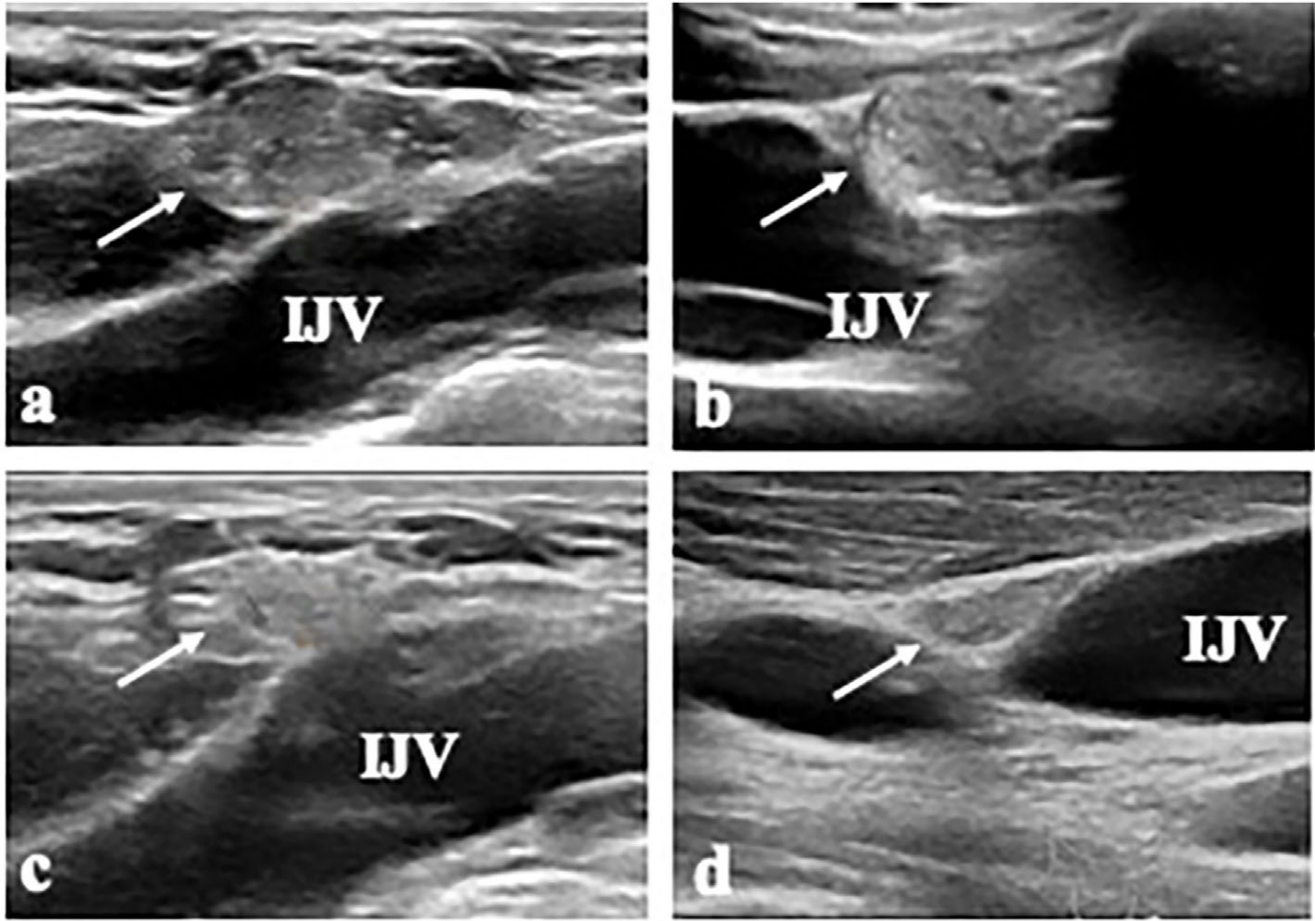
A 17-year-old male patient (Patient 4) with metastatic LN (LN 7) at left level IV. **(A, B)** The transverse and longitudinal US shows a 2.0-cm-sized hypoechoic mass (arrow) near the internal jugular vein (IJV) at left level IV. **(C, D)** At 15 months after RFA, the transverse and longitudinal US shows the ablated LN is decreased in size (arrow).

The outcomes of RFA for metastatic LNs in patients with PTC are summarized in **Table 2**. The mean follow-up time was 52.00 ± 21.44 months (range 15-70 months). The mean largest diameter of the metastatic LNs decreased significantly from 1.16 ± 0.52 cm to 0.07 ± 0.22 cm(*P*=0.005). The mean volume of the metastatic LNs decreased significantly from 0.30 ± 0.38 ml (range 0.06-1.23 ml) to 0.01 ± 0.03 ml (range 0-0.09ml) (*P*=0.005) with a mean VRR of 99.28 ± 2.27% (range 92.82-100%) (**Figures 1** and **2**). A total of 9 metastatic LNs (90.00%) completely disappeared at the last follow-up. The Tg level was decreased from 25.10 ± 15.20 ng/mL (range 11.40-43.40 ng/mL) to 12.27 ± 11.96 ng/mL (range 0.03-30.20 ng/mL) (*P*=0.042).

**Table 2:** Outcomes of RFA for metastatic LNs in pediatric patients with PTC.

No. of patient	No. of LN	Initial Volume (ml)	Follow-up time/Complete disappearance (months)	Volume at last follow-up (ml)	Recurrence after RFA/interval time (months)	Treatment for recurrence after RFA
1	1	0.06	62/6	0	–	–
	2	0.06	62/6	0		
2	3	0.35	56/3	0	–	–
	4	0.08	56/3	0		
3	5	0.68	70/8	0	Multiple LN metastases/24	Total thyroidectomy with central neck dissection
	6	0.06	70/8	0		
4	7	1.23	15/-	0.09	One metastatic LN in the right cervical level IV/15	RFA
	8	0.28	15/15	0		
	9	0.07	15/15	0		
5	10	0.16	58/6	0	–	–

After RFA, there was no evidence of recurrence at the initial treatment location. A total of 2 patients developed newly metastases. Patient 3 had multiple LN metastases at 2 years after RFA. This patient underwent total thyroidectomy with central neck dissection. During the surgery, no adhesion with strap muscle by RFA was found. After histopathological examination, metastases were detected in 8 out of 10 resected LNs. Patient 4 had one newly metastatic LN (largest diameter 1.3cm, volume 0.44ml) at right level IV at 15 months after RFA and underwent additional RFA. A power of 6W was used. The ablation time was 77s, and the energy was 390J. This newly metastatic LN was ablated successfully with no complications.

## Discussion

This study is the first to demonstrate the efficacy and safety of RFA for cervical metastatic LNs in children and adolescents with PTC. Our study showed that after a mean follow-up time of 52.00 ± 21.44 months, the VRR of metastatic LNs was 99.28 ± 2.27% and 90.00% of the ablated LNs completely disappeared. After RFA, a total of two patients developed LN metastases. One patient underwent total thyroidectomy with central neck dissection and the other one chose additional RFA. All the patients were well tolerable the RFA procedure. No complications were encountered. These results showed that RFA might be effectively applied in children and adolescents without increasing technical difficulties by former surgery.

The incidence of pediatric DTC has been rising in recent years and PTC accounts for over 90% (2). The overall survival remains excellent with a low rate of mortality even in advanced stage, however, pediatric patients have a high rate of recurrence (2, 22). Perry et al. (23) reported that with a median follow-up time of 11 years, 34% of the children and adolescents experienced a recurrence and the mean time to first recurrence of disease was 5.3 years. Among them, 50% were LN metastases and 29% were lung metastases. Hay et al. (3) showed that by 40-year follow-up for 215 children and adolescents with PTC, 32% of patients had experienced a recurrence within the neck or at distant sites. Moreover, 73% of the first postoperative recurrences had been localized to the regional neck nodes. Spinelli et al. (22) found that multifocality, vascular invasion, infiltration of the thyroid capsule, minimal extrathyroidal extension, diffuse sclerosing variant of PTC and present of LN metastases in the lateral compartment were significantly associated with LN metastases in central compartment in children and adolescents. The predictive factors for LN metastases in lateral compartment were infiltration of the thyroid capsule, massive extrathyroidal extension, distant metastases, PTC, and the presence of LN metastases in the central compartment. Pediatric patients with those predictors might need a stricter follow up after PTC diagnosis and treatment. According to the ATA guideline, the decision to treat or to observe structurally identifiable cervical recurrent disease should be individualized (2). Repeat surgery was recommended for cervical disease > 1cm in size (2). However, the scar formation and normal tissue plane distortion by former surgery could bring technical challenges for repeat surgery (1, 24). It could also cause psychological trauma and serious complications, including hypoparathyroidism, recurrent laryngeal nerve damage and Horner syndrome (1, 2, 25). For patients with small-volume cervical disease, there were two options. One was observation with TSH suppression. However, there were no data in pediatric patients with which to compare the potential benefits with the potential risks of various TSH suppression strategies (2). The other one was ^131^I therapy, which might reduce future recurrence risk but was unlikely to improve mortality (2). Given the increased risks of secondary malignancies and complications including short-term side effects and delayed toxicities, the use and dosage of ^131^I therapy should be chosen in a thoughtful manner to avoid potential disease far worse than the one being treated (26–28). In addition, some patients were not suitable for ^131^I therapy, because they only underwent thyroid lobectomy, not total thyroidectomy. Therefore, finding an alternative less invasive than repeat surgery and ^131^I therapy might be helpful.

RFA, as a minimally invasive technique, can induce irreversible cell injury and ultimately tumor apoptosis and coagulative necrosis (29). It has been recommended as a safe and effective treatment for benign thyroid nodules and recurrent thyroid cancers in adults (10). However, its application in children and adolescents with thyroid disease was uncertain. Recently, Hong et al. (30) reported the application of RFA for nonfunctioning benign thyroid nodules in 14 children and adolescents. During a mean follow-up time of 36.9 ± 21.7 months, the volume was decreased from 14.6 ± 13.1 ml to 1.7 ± 4.4 ml with a mean VRR of 92.1 ± 11.4%. Both cosmetic and compressive symptoms improved significantly. It suggested that RFA had potential application in cervical thyroid-related disease of children and adolescents. This study showed that after a mean follow-up time of 52.00 ± 21.44 months, the VRR of metastatic LNs was 99.28 ± 2.27% and 90.00% of the ablated LNs completely disappeared. Although the object of this study was children and adolescents, similar results were observed in adults. Guang et al. (31) demonstrated that with a mean follow-up time of 21 ± 4 months after RFA, the VRR was 94.9 ± 5.3% and 61.1% of the metastatic LNs completely disappeared. Chung et al. (32) reported after a long-term follow-up time of 80 ± 17.3 months, the volume of metastatic LNs decreased from 0.25 ± 0.42ml to 0.01 ± 0.08ml with a VRR of 99.5 ± 2.9% and the complete disappearance rate was 91.3%. These results indicated that the efficacy of RFA could not be affected by the age of patients.

The complications of RFA was various, but none of them was life-threatening. Some patients only had various degrees of pain or a sensation of heat during RFA for treating benign thyroid nodules (30). In this study, all the patients were tolerant to RFA procedure and no procedure-related complications occurred. We used three treatment strategies to reduce the rate of complications. First, the RFA procedure was performed by an experienced US physician. It was very important because the anatomical structures of pediatric patients were not developed completely and were distorted by former surgery. Second, in order to prevent thermal injury, hydrodissection technique was used to separate the target LN from critical structures. Meanwhile, real-time US imaging could also allow the physician to monitor the RFA electrode tip and adjacent critical structures (10). Third, careful lidocaine injection around the target LN could reduce the patient’s pain and allow sufficient ablation. Furthermore, as a minimally invasive technique, RFA only needed local anesthesia in the outpatient department without scars and hospitalization, which could avoid operative-related risks and relieve long-term distress and psychological trauma in children and adolescents.

The recurrence rate after RFA for adults with metastatic LNs was from 1.9% to 12.5% (24, 31, 33, 34), however, the result in this study was relatively high. Similarly, previous studies reported the recurrence rate was 33-60% after RFA for solid tumors in pediatric patients (11, 14, 15, 19). There were two possible explanations. First, the incidence of PTC recurrence was much lower in adults than in pediatric patients (2). Bilimoria et al. (35) published a series of 52,174 adult patients with PTC from the National Cancer Database and found that the overall recurrence rates were 5.7% at 5 years and 9.4% at 10 years. In another study of 1088 adult patients with PTC, the recurrence rate was 4.8% at a median follow-up period of 17.6 years (36). However, the recurrence rates of pediatric PTC at 5, 10, 20 and 30 years were 20%, 22%, 27%, and 30%, respectively (3). Second, the risk of recurrence disease from pediatric PTC was associated with the initial surgical approach. Hay et al. (3) reported that during 40-year follow-up, the recurrence rates after bilateral lobar resection and lobectomy were 25% and 65%, respectively (3). The local and regional recurrence rates after lobectomy (35%, 60%) were significantly higher than after bilateral lobar resection (6%, 13%) (3). In this study, most patients (3/5) did not have total thyroidectomy and they may have a higher underlying burden of disease and an increased risk for recurrence. During the follow-up of RFA, a total of two patients developed newly metastases. One patient chose additional RFA. The other one underwent total thyroidectomy with central neck dissection and multiple microscopic metastases were found after histopathological examination. Because the sensitivity of US to detect central metastatic LN and microscopic metastases was low, malignancy could only be confirmed after surgical dissection. Therefore, repeat surgery was undoubtedly a definitive curative treatment for children and adolescence. However, RFA could be used as a safe and effective local control of cervical metastatic LNs and may provide another alternative to the existing therapeutic modalities for children and adolescents with multiple surgery or surgical ineligibility.

This study has some limitations. First, it was a single-center retrospective study. Further prospective multicenter studies are needed. Second, the number of cases included was small and the follow-up period was relatively short. Considering the rarity of pediatric PTC, it was difficult to accumulate more cases. According to a systematic review of ablation techniques for children, only 28 patients were identified to be treated by ablation (19). Based on the available data, this study seemed to have an acceptable sample size to summarize and evaluate our experience.

## Conclusion

As a less invasive and effective technique, RFA may provide another alternative to the existing therapeutic modalities for cervical metastatic LNs in children and adolescents with PTC.

## Data Availability Statement

The datasets presented in this article are not readily available because of patients’ privacy. Requests to access the datasets should be directed to gemma-y@163.com.

## Ethics Statement

The studies involving human participants were reviewed and approved by Institutional Review Board of Chinese PLA General Hospital. Written informed consent to participate in this study was provided by the participants’ legal guardian/next of kin.

## Author Contributions

LY interpreted the patient data and drafted the manuscript. YKL performed RFA procedure, conceived of the study and coordination. YZ and JB collected and analyzed the patient data. All authors contributed to the article and approved the submitted version.

## Funding

This study is supported by Beijing Municipal Science & Technology Commission (No. Z181100001718017), National Natural Science Foundation of China (No. 81771834), and the Research of Healthcare Big Data f Chinese PLA General Hospital (2019MBD-040).

## Conflict of Interest

The authors declare that the research was conducted in the absence of any commercial or financial relationships that could be construed as a potential conflict of interest.
